# Diversity and Emerging Roles of Enhancer RNA in Regulation of Gene Expression and Cell Fate

**DOI:** 10.3389/fcell.2019.00377

**Published:** 2020-01-14

**Authors:** Preston R. Arnold, Andrew D. Wells, Xian C. Li

**Affiliations:** ^1^Texas A&M Health Science Center, College of Medicine, Bryan, TX, United States; ^2^Immunobiology and Transplant Sciences, Department of Surgery, Houston Methodist Hospital, Houston, TX, United States; ^3^Department of Pathology and Laboratory Medicine, Perelman School of Medicine, University of Pennsylvania, Philadelphia, PA, United States

**Keywords:** eRNA, enhancer, super enhancer, phase separation, gene regulation, non-coding RNA

## Abstract

Enhancers are *cis*-regulatory elements in the genome that cooperate with promoters to control target gene transcription. Unlike promoters, enhancers are not necessarily adjacent to target genes and can exert their functions regardless of enhancer orientations, positions and spatial segregations from target genes. Thus, for a long time, the question as to how enhancers act in a temporal and spatial manner attracted considerable attention. The recent discovery that enhancers are also abundantly transcribed raises interesting questions about the exact roles of enhancer RNA (eRNA) in gene regulation. In this review, we highlight the process of enhancer transcription and the diverse features of eRNA. We review eRNA functions, which include enhancer-promoter looping, chromatin modifying, and transcription regulating. As eRNA are transcribed from active enhancers, they exhibit tissue and lineage specificity, and serve as markers of cell state and function. Finally, we discuss the unique relationship between eRNA and super enhancers in phase separation wherein eRNA may contribute significantly to cell fate decisions.

## Introduction

Enhancers are short regulatory elements of accessible DNA that help establish the transcriptional program of cells by increasing transcription of target genes. They are bound by transcription factors, co-regulators, and RNA polymerase II (RNAP II). Enhancers are flanked by histones with permissive chromatin markers, including H3K4 methylation (H3K4me) and H3K27 acetylation H3K27ac). While promoters direct gene transcription in a position- and orientation-dependent manner, enhancers traditionally function independently of their position and orientation with respect to their target gene, as they can loop over long genomic ranges to engage distant promoters (Kim et al., 2015). Despite the fundamental role of enhancers in cellular biology, we are still learning about the mechanisms by which they promote gene transcription, and consequently, cell fate decisions.

A surprising discovery regarding enhancers was made in 2010, when it was shown that enhancer regions are actively transcribed (De Santa et al., 2010; Kim et al., 2010). The product of this transcription, termed “enhancer RNA” or “eRNA,” has subsequently been the source of great debate and speculation. While pervasively observed, the role of most eRNAs has remained enigmatic. This has lead many to suggest that enhancer transcription is the noisy byproduct of transcription machinery. However, a growing number of studies suggests diverse roles for eRNA in regulating many aspects of cell functions. Notwithstanding, it remains unclear whether eRNA which functions are generalizable. It is also unclear how eRNA structure is related to its functional relationships in complex gene regulation machinery.

While enhancers are critical to all cell types, eRNA abundance varies dramatically across tissues. The FANTOM5 project identified immune cells, neural tissues, and hepatocytes among those with the highest abundance of cell-specific enhancers, a higher ratio of enhancers/gene, and high enhancer transcription (Andersson et al., 2014a). Indeed, studies in neurons, macrophages, and other immune cells are prominent among those contributing to our understanding of enhancers and eRNA. In contrast, smooth muscle, fibroblasts, and epithelial cells tend to utilize enhancers with less cell-specificity and have a lower enhancer/gene ratio (Andersson et al., 2014a). This diversity of enhancer utilization likely reflects the unique roles and needs of each cell type to dynamically respond to its environment.

In this review, we outline current understanding of eRNA production, structure, and classification, and discuss recent findings concerning eRNAs in gene regulation. Finally, we discuss super-enhancers, their relationship with eRNA, and the potential roles of eRNA in super-enhancer formation and structure, as well as their roles in phase separation and cell fate decisions.

## Cellular Production of eRNA

The genome-wide transcription of enhancer regions by RNAP II was first reported by Kim et al. (2010) in neurons and by De Santa et al. (2010) in macrophages. This transcription is accompanied by the presence of both general transcription factors and lineage-specific transcription factors at the enhancer loci (Koch et al., 2011; Hah et al., 2013; Kaikkonen et al., 2013; Pulakanti et al., 2013; Cauchy et al., 2016). Extracellular stimuli drive signaling pathways which remodel chromatin and alter eRNA and mRNA expression (Kaikkonen et al., 2013; Heward et al., 2015). eRNA transcription occurs very early in the gene transcription process, preceding mRNA expression from adjacent *cis* loci (Schaukowitch et al., 2014; Arner et al., 2015; Kim et al., 2015; Baillie et al., 2017; Shii et al., 2017; Tyssowski et al., 2018). Indeed, the ubiquitous and early production of eRNAs makes them an excellent marker for segregating active versus quiescent enhancers, which is discussed in greater detail below (Andersson et al., 2014a; Tyssowski et al., 2018).

**FIGURE 1:**
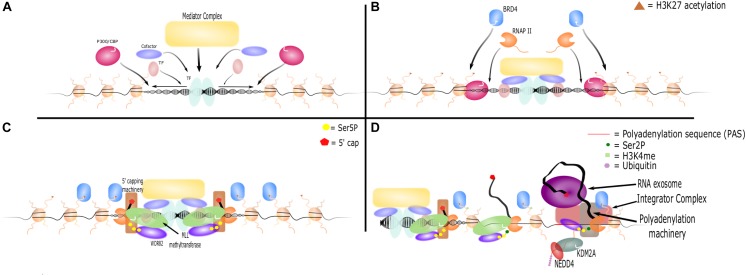
eRNA transcription, elongation, and termination. **(A)** Activated transcription factors bind the enhancer locus and promote nucleosome remodeling. They recruit other transcription factors, cofactors, complexes such as Mediator, and histone modifiers such as P300/CBP. **(B)** P300/CBP acetylates H3K27, which further opens the enhancer locus and recruits additional proteins such as BRD4 and RNAP II. **(C)** The CTD of RNAP II is phosphorylated at Ser5. WDR82 binds and recruits MLL methyltransferases, which act as cofactors to initiate RNA transcription. 5′ capping machinery is also recruited to Ser5P and caps the nascent RNA strand. **(D)** BRD4 and other cofactors facilitate RNAP II transition to elongation, which results in an increase in Ser2P marks on the CTD of RNAP II and methylation of H3K4. However, PASs shortly downstream of the TSS are recognized by WDR82. This leads to recruitment of polyadenylation machinery and Integrator to terminate RNA transcription. Additionally, the RNA exosome is recruited and binds the 5′ cap, leading to rapid degradation of RNA. KDM2A recruits NEDD4 to RNAP II leading to its ubiquitination and dismissal.

The process of eRNA production proceeds through the following steps: (1) recruitment of transcription factors and coactivators necessary for enhancer formation and gene activation, (2) histone modifications at the active enhancer loci, and (3) eRNA transcription, elongation, and processing (Kaikkonen et al., 2013; Figure 1).

Stimulus-dependent and/or cell type-specific transcription factor and cofactor binding at specific sites in the genome are critical first steps to the formation and activation of enhancers (Kaikkonen et al., 2013; Arner et al., 2015). In macrophages, multiple signaling pathways and transcription factors induce a pro-inflammatory gene program and eRNA transcription in response to LPS (Heward et al., 2015). In some cases transcription factors bind to constitutively accessible DNA at existing enhancer regions, while in other cases *de novo* nucleosome remodeling precedes enhancer activation and eRNA transcription (Kaikkonen et al., 2013; Ortega et al., 2018). Specific histones, such as H2A.Z, may be necessary for the loosening of DNA from nucleosomes at enhancer regions. Such loosening allows transcription factors to bind and enact enhancer functions (Brunelle et al., 2015). Once bound, transcription factors recruit coactivators and other complexes of DNA binding proteins (such as the MegaTrans complex in breast cancer cells) that are necessary for enhancer activation, eRNA transcription, and gene trans-activation (Liu et al., 2014). Thus, transcription factor binding with subsequent recruitment of additional cofactors and complexes provides the initial framework for enhancer activation and eRNA transcription.

Transcription at enhancer regions is dependent on epigenetic modification of histones and DNA. These processes are mediated by histone modifying and DNA demethylating components of coactivator complexes (Stadler et al., 2011; García-González et al., 2016), with H3K27ac and H3K4me1/2 being the most pervasively associated with actively transcribed enhancers (Djebali et al., 2012). H4K8 and H3K27 acetylation by the p300 and CBP histone acetyltransferases occurs following transcription factor binding and concordantly with eRNA production (Kaikkonen et al., 2013). Inhibition of their acetyltransferase activity results in concomitant loss of H3K27ac, RNAP II occupancy, eRNA transcription, and enhancer function (Jiao et al., 2018; Raisner et al., 2018). The H3K27me2/3 demethylase Kdm6a/b was also shown to act as a coactivator of eRNA transcription (Li et al., 2017; Kyzar et al., 2019). Conversely, recruitment of repressive Polycomb H3K27 methyltransferases or histone deacetylases (HDAC) have been shown to decrease eRNA expression (Cao et al., 2002; Kaikkonen et al., 2013; Lam et al., 2013). The dependence of eRNA transcription on histone acetylation is due in part to the recruitment of Bromodomain-Containing Protein 4 (BRD4) to enhancer regions by di-acetylated nucleosomes. BRD4 facilitates RNAP II recruitment and elongation through hyperacetylated enhancer regions (Kanno et al., 2014). BET inhibitors, which interfere with the ability of BRD4 bromodomains to interact with acetylated histones, also inhibit eRNA transcription (Kanno et al., 2014; Rahnamoun et al., 2018). Thus, histone acetylation not only marks enhancer sites but is crucial for eRNA production and enhancer function, likely through BRD4-dependent mechanisms.

In addition to histone modifications, enhancers exhibit decreased DNA methylation (Stadler et al., 2011), and actively transcribed enhancers show decreased DNA methylation compared to non-transcribed enhancers (Pulakanti et al., 2013). In mouse embryonic stem cells, these hypomethylated, actively transcribed regions were occupied by TET family DNA hydroxylases, which can actively demethylate CpG dinucleotides (Stadler et al., 2011). In a liver cancer model, hypomethylated DNA was shown to be necessary for eRNA production as targeted DNA demethylation through a Cas9-TET1 fusion increased eRNA production (Xiong et al., 2019). In another study, the knockdown of TET1 and TET2 decreased eRNA production at some enhancers but not others (Pulakanti et al., 2013). Therefore, while DNA demethylation appears to be important for enhancer activation and transcription, the mechanism and role of DNA demethylation and TET enzymes in eRNA production remain uncertain.

The details and mechanisms of enhancer transcription, initiation, and elongation are similar to those found at promoter sites, and the similarities between eRNA, lncRNA, PROMPT, and mRNA transcription have been thoroughly reviewed (Li et al., 2016). Critical to eRNA transcription is the phosphorylation of the C-terminal domain (CTD) of RNAP II and an abundance of poly(A) signals (PAS) immediately downstream of the transcription start site (TSS). Phosphorylation of Tyr1 residues on the CTD is associated with enhancer transcription and RNAP II stability (Descostes et al., 2014). RNAP II at enhancers has high levels of Ser5P relative to Ser2P on its CTD, indicative that it is not undergoing prolonged elongation (Koch et al., 2011; Pulakanti et al., 2013). This is likely due to the abundance of PAS which are bi-directionally found immediately downstream of enhancer TSSs (Ntini et al., 2013; Andersson et al., 2014a, b; Austenaa et al., 2015). Polyadenylation of eRNA has been shown to promote exosome recruitment, RNAP II instability, and transcription termination (Henriques et al., 2018). The adaptor protein WDR82 is important for RNAP II recognition of PAS and is necessary for transcription termination and cleavage of short RNA molecules such as PROMPTs and eRNA (Austenaa et al., 2015). Similarly, the INTS9 and INTS11 subunits of the Integrator complex contain nuclease activity necessary for cleaving snRNAs and eRNAs from RNAPII (Lai et al., 2015; Skaar et al., 2015). Loss of WDR82 or Integrator prevented cleavage of eRNA transcripts from RNAP II, leading to unusually long eRNAs and accumulation of unprocessed primary eRNA transcripts. These studies demonstrate the importance of WDR82 and Integrator to transcriptional termination at enhancers and eRNA maturation (Austenaa et al., 2015; Lai et al., 2015).

Interestingly, Integrator and WDR82 are involved in both the preparatory and terminating steps of eRNA transcription. WDR82 also plays a role in recruiting MLL methyltransferases to enhancers (Austenaa et al., 2015). These methyltransferases methylate H3K4 to H3K4me1/2 during transcription and have also been shown to act as coactivators of eRNA transcription independently of their methyltransferase activity (Dorighi et al., 2017). In macrophages, the INTS13 subunit of Integrator interacts with the EGR1/2 transcription factors and the NAB2 cofactor to bind and activate poised enhancers (Barbieri et al., 2018). This biological coupling of eRNA initiation and termination suggests careful regulation of eRNA and implicates eRNA molecules as more than simple accidental transcriptional noise.

## Structure and Heterogeneity of eRNA

Enhancer RNAs represent a diverse class of molecules in terms of their structure and transcription patterns. Originally described as non-polyadenylated, bidirectionally transcribed RNA transcripts of less than 2 kb that originate from active enhancers marked by H3K4me1 (Kim et al., 2010), it was subsequently shown that some eRNAs can be polyadenylated or unidirectionally transcribed. Polyadenylated eRNAs are frequently longer (up to 4 kb), unidirectionally transcribed, and transcribed from higher-activity enhancers than are their bidirectional, non-polyadenylated counterparts (Koch et al., 2011). The genome-wide FANTOM5 enhancer atlas largely corroborated these distinctions, showing that the majority of eRNAs are short (median of 346 nucleotides), bidirectionally transcribed, unspliced, and non-polyadenylated (Andersson et al., 2014a). However, incredible heterogeneity has been noted among all structural aspects of eRNAs. Nearly all combinations of transcriptional directionality, length, splicing, and polyadenylation have been reported (Hsieh et al., 2014; Alvarez-Dominguez et al., 2017; Jiao et al., 2018). Many enhancers produce eRNA bidirectionally but with the predominant production coming from either the negative or positive strand (Pulakanti et al., 2013; Andersson et al., 2014b; Schaukowitch et al., 2014). In at least one report, it was shown that the dominantly transcribed eRNA also provided the majority of biological function ascribed to eRNA from that enhancer (Hsieh et al., 2014). Additionally, single-cell CAGE sequencing revealed that while on a bulk level enhancers can be described as bi-directionally transcribed, on a single-cell level enhancers are almost exclusively unidirectionally transcribed from either strand (Kouno et al., 2019). This observation complicates the absolute classification into bidirectionally or unidirectionally transcribed. Thus, the directionality of enhancer transcription is more complex than “unidirectional” or “bidirectional” and truly exists along a gradient of strand transcriptional preference.

The polyadenylation of eRNA is not universal (Djebali et al., 2012), perhaps due to active 3′ degradation of eRNAs (Pefanis et al., 2015). The nuclear RNA exosome is known to degrade the 3′ end of eRNAs (Andersson et al., 2014a, b; Pefanis et al., 2015; Imamura et al., 2018). Because the distance between TSS and PAS is inversely correlated with RNA exosome sensitivity, it has been speculated that close proximity between the TSS and the PAS prevents the complete assembly of polyadenylation machinery on the C-terminal-domain of RNAP II. This leads to suboptimal polyadenylation, RNA exosome intervention, and rapid degradation (Ntini et al., 2013). Such a model would explain why longer eRNAs tend to be polyadenylated, and is supported by studies that have examined specific eRNA length, polyadenylation, and degradation (Schaukowitch et al., 2014; Tsai et al., 2018). Additional studies have found that the same eRNAs can be detected through RT-PCR using oligo-dT or random hexamer primers, with the oligo-dT primers generally providing weaker signal (Hah et al., 2013; Pulakanti et al., 2013). This matches eRNA transcription termination studies wherein shorter eRNAs are sub-optimally polyadenylated and subjected to rapid degradation.

In addition to primary structure heterogeneity, eRNAs demonstrate diverse secondary structures that model those seen in tRNAs, miRNAs, snRNAs, and lncRNAs (Cheng et al., 2015; Ren et al., 2017). Some eRNA molecules contain different domains with unique functions (Cajigas et al., 2018; Cichewicz et al., 2018; Tsai et al., 2018). Post-transcriptional modifications have also been observed, such as cytosine methylation by NSun7, which may promote stability of eRNAs (Aguilo et al., 2016). Another study found enrichment of *N*^6^-methyladenosine (m^6^A) among enhancer-derived long intergenic non-coding RNAs (e-lincRNAs), which plays roles in RNA structure, stability, and processing (Xiao et al., 2019). Thus, the true diversity of eRNAs is not captured in its lengths or directionality of transcription, but in the various biological functions its secondary structures and post-transcriptional modifications portend.

An important issue for eRNA classification is the relationship between eRNA and lncRNAs. Long polyadenylated eRNAs are structurally similar to lncRNAs, but are transcribed from active enhancers rather than promoters. However, whereas much of our understanding of promoters comes from coding genes, many promoters do not fit the classic TATA structure. The rigidity of a binary promoter/enhancer paradigm blurs outside the context of coding genes, and it has been shown that some promoters may act as enhancers depending on the context (Mikhaylichenko et al., 2018; Rennie et al., 2018). Our emerging understanding may therefore unify long polyadenylated eRNAs and lncRNAs into a single class of regulatory RNAs transcribed from regulatory regions. Continuous re-evaluation of eRNA classification and grouping will be necessary as our understanding and definition of enhancer regions and non-coding RNAs continues to evolve.

Taken together, the above studies highlight that eRNAs are a heterogeneous and, as yet, poorly defined group of molecules. This diversity certainly leads to distinct biological functions, which will be discussed below, as we review the functional roles for eRNAs in gene regulation.

## Biological Functions of eRNA

### eRNA as Regulators of Gene Expression

A central issue regarding our current understanding of eRNA is whether the transcription of eRNA or the eRNA itself is primarily responsible for observed fuctnions. For example, active transcription at enhancer sites can alter chromatin architecture and epigenetics and can recruit specific proteins to the enhancers (Kaikkonen et al., 2013; Meng et al., 2014; Benner et al., 2015). Additionally, antisense transcription of intragenic enhancers may play a role in attenuating and fine-tuning gene transcription by interfering with or pausing the sense mRNA transcription (Cinghu et al., 2017). Thus, transcription at enhancers may be correlated with significant biological phenomena without the produced eRNA playing a direct role in that phenomena.

Nevertheless, emerging evidence implicates a role for eRNA itself in transcription regulation. On a genomic scale, eRNA transcription and induction of mRNA transcription at neighboring genes are correlated (Kim et al., 2010; Kaikkonen et al., 2013; Li et al., 2013; Andersson et al., 2014a; Arner et al., 2015; Cheng et al., 2015; Dorighi et al., 2017; Ren et al., 2017; Imamura et al., 2018). RNAP II is not only abundantly present at active enhancers, but appears to be critical to their function as ubiquitination and dismissal of RNAP II leads to enhancer decommission and downregulation of target genes (Tan et al., 2018). eRNA knockdown studies have demonstrated important roles for individual eRNAs in the regulation of their target genes (Supplementary Table 1). Additional studies have shown that exogenous overexpression of eRNAs leads to an increase in their respective mRNA targets (Alvarez-Dominguez et al., 2017; Shii et al., 2017; Jiao et al., 2018). These studies have provided the bulk of evidence implicating a directly causal role for eRNA, rather than the act of transcription, in the association between enhancer and mRNA transcription. However, it remains unclear how generalizable these instances are across the tens of thousands of enhancers known to transcribe eRNAs. Thus, the relevant question is not *if* eRNAs have a biological role, but *which* eRNAs are functional, and how eRNA function is linked to structure and localization.

Enhancer RNAs appear to be specific for their putative targets. This is based on the observation of strong correlation between eRNA transcription and neighboring mRNA transcription, as well as in knockdown experiments that show selective decrease in corresponding mRNAs when specific eRNAs are knocked down (Supplementary Table 1) (Arner et al., 2015). It is generally presumed that such specificity is largely context-dependent and driven by proximity and other factors controlling enhancer-promoter interactions (see van Arensbergen et al. (2014) for review of enhancer-promoter specificity). However, ectopically expressed Bloodlinc and SERPINB2 eRNAs have also shown the ability to selectively upregulate their respective mRNAs suggesting that, at least among these eRNAs, mere proximity is insufficient to explain their specificity for their targets (Alvarez-Dominguez et al., 2017; Shii et al., 2017). Several studies utilizing reporter assays to investigate how eRNAs promote transcription at their respective mRNA promoter have demonstrated the importance of the specific eRNA sequence in promoting transcription compared to truncated or “missense” RNA transcripts (Lam et al., 2013; Li et al., 2013; Melo et al., 2013). One such study observed that eRNA known to upregulate a different mRNA did not increase expression in their reporter, suggesting specificity of eRNA-promoter interactions (Aguilo et al., 2016). These studies suggest there are specific features (such as sequence or secondary structures) that confer eRNA function and that such traits may even provide for specific eRNA-promoter interactions. The traits and mechanisms for such intrinsic specificity remain elusive, but could include interactions with protein complexes, base-pairing with target DNA, or other undefined mechanisms.

### Mechanisms of Action

**FIGURE 2:**
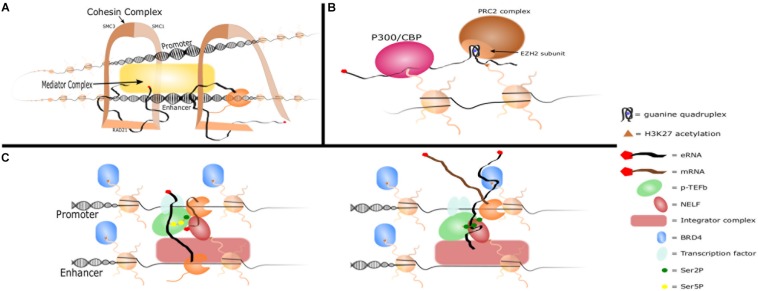
eRNA mechansims of action. **(A)** Promoter-enhancer looping. eRNA interacts with Cohesin complex subunits RAD21 and SMC3 to recruit them to the enhancer locus and promote enhancer-promoter looping. eRNA also interacts with other complexes such as Mediator to promote looping. **(B)** Histone modifications. eRNA increases H3K27ac by binding P300/CBP and promoting its affinity for H3K27. eRNA also inhibits the H3K27me complex PRC2 by binding the EZH2 subunit with a guanine quadruplex. **(C)** Interactions with transcriptional machinery. eRNA is brought into close contact with NELF and paused RNAP II by Integrator complex. It interacts with the CDK9 subunit of p-TEFb which phosphorylates NELF and Ser2 of RNAP II’s CTD and increases the affinity of transcription factors for DNA. It then replaces the nascent mRNA strand in binding NELF-E, which allows RNAP II escape and transition to productive elongation. eRNA also increases affinity of BRD4 for acetylated histones.

In spite of the abundance of evidence supporting eRNAs as functional biomolecules, the exact mechanism by which they promote gene transcription remains enigmatic. However, several potential mechanisms of action are emerging including promotion of enhancer-promoter interactions, chromatin modifications, or regulation of transcriptional machinery (Figure 2). These potential mechanisms are not mutually exclusive and are effected through interactions with nuclear proteins. For example, ^DRR^eRNA and Bloodlinc eRNA were shown by mass spectrometry to bind to over 30 proteins, many of which have known functions in chromatin remodeling and gene regulation (Alvarez-Dominguez et al., 2017; Tsai et al., 2018). eRNA-protein interactions may play roles in protein recruitment, altering protein interactions, and providing scaffolding. An example is the cooperation of eRNA with YY1, a transcription factor with both DNA and RNA binding activity. RNAs tethered near enhancer loci were shown to increase the presence of YY1 specifically at those loci, perhaps due to “transcription factor trapping” wherein nascent RNA from enhancers and promoters increase the affinity of otherwise weak DNA-TF interactions creating a kinetic sink that would “trap” escaped transcription factors (Sigova et al., 2015). Such a model may explain observations that eRNA increases the binding of transcription factors such as c-Jun and NF-κB to target loci (Shii et al., 2017; Spurlock et al., 2017; Huang Z. et al., 2018). An emerging idea for how eRNAs may interact with large numbers of proteins is through phase separation. Phase separation describes the large-scale, three-dimensional concentration of proteins and nucleic acids (Box 1) and is a means by which transcriptional machinery and multiple regulatory elements are brought together. Phase separation of enhancers has been shown at super enhancers and at enhancers utilizing the MegaTrans complex (Sabari et al., 2018; Nair et al., 2019). While much remains to be learned, the literature overall supports a model wherein eRNAs contribute to enhancer function by interacting with nuclear proteins to promote enhancer-promoter looping, chromatin modification, and regulation of transcriptional machinery, possibly through phase separation.

**BOX 1. Elements of Phase separation**

Phase separation leads to the formation of biomolecular condensates, which are membraneless structures in the cell. The unique chemical and physical properties of certain proteins and nucleic acids cause them to polymerize, decreasing their solubility and allowing them to form their own liquid phase separate from the cytoplasm (Banani et al., 2017). This forms a unique microenvironment which selectively recruits and concentrates similar proteins and molecules while excluding others: analogous to oil and water except that participating molecules pass freely between the condensate and cytoplasmic phases. Condensates function to organize and sequester cellular resources, regulate specific reactions, and alter reaction kinetics (Banani et al., 2017). These structures are pervasive throughout cellular biology and examples include nucleoli, stress granules, Cajal bodies, paraspeckles, P bodies, and membrane clusters (Nott et al., 2015; Saha et al., 2016; Fox et al., 2018). Biomolecular condensates are made of both “scaffolding” molecules which contribute to their initial formation and “client” molecules which affect their composition and chemistry. Additionally, biomolecular condensates are heterogeneous structures that display different “layers” and subcompartments that are driven by unique protein-protein, protein-RNA, and RNA-RNA interactions (Boeynaems et al., 2019). A primary driver of phase separation are proteins with large intrinsically disordered regions (IDR). IDR-mediated phase separation is especially common among condensates that concentrate RNA (Banani et al., 2017). IDRs are critical components of RNA binding domains (RBD) that mediate the phase separation of their constitutive proteins (Lin et al., 2015; Zhang et al., 2015; Wang et al., 2018). RNA binding alters the IDR’s physical properties, and accordingly, RNA structure and concentration are critical to driving or preventing the formation of many condensates as a scaffolding molecule (Lin et al., 2015; Zhang et al., 2015; Banani et al., 2016; Saha et al., 2016; Langdon et al., 2018; Maharana et al., 2018; Ries et al., 2019). RNA is also an important client contributing to the maintenance and composition of biomolecular condensates. RNA both recruits components to the condensates and helps establish the unique composition of proteins within different phase separated structures. Altering the stoichiometry of scaffolding RNAs or proteins can change which proteins are recruited to the condensate (Banani et al., 2016; Langdon et al., 2018). RNA has also been shown to affect biophysical characteristics of condensates such as viscosity, dynamics of component exchange with the non-condensate solution, and ability to fuse with other condensates (Zhang et al., 2015). Phase separated structures, especially those containing RBD/IDR domains, are known to “mature”: a process wherein they become increasingly less liquid and display more solid-like properties (Lin et al., 2015; Molliex et al., 2015; Zhang et al., 2015; Banani et al., 2017). As with formation, the impact of RNA on each of these areas is mediated by secondary structure and concentration (Langdon et al., 2018; Maharana et al., 2018). Thus, RNA is a key contributor to the biology of biomolecular condensates. For an extensive review on phase-separation and biomolecular condensates, please see Banani et al. (2017).

#### Enhancer-Promoter Interaction

The traditional mode of action by active enhancers in regulating distant target genes is through chromatin looping to engage the gene promoters. Shortly after their discovery, it was noted that eRNAs were prominent at looped enhancers, but whether eRNA played a role in that looping process was less clear (Wang et al., 2011; Sanyal et al., 2012). It has been shown that eRNA transcription and chromatin looping occur concomitantly at the β-globin locus control region, which precedes gene transcription (Kim et al., 2015). The first study to propose enhancer-promoter interactions as a potential role for eRNA function was conducted by Li et al. (2013). Using chromosomal conformation capture analysis and siRNA knockdown to investigate changes in eRNA-enhancer-promoter interactions in response to estrogen stimulation, they observed that the knockdown of eRNAs resulted in decreased interactions between the enhancer and its nearest promoter. These studies found through *in vitro* transcribed (IVT) RNA-pulldown, RNA immunoprecipitation (RIP), and RIP-qPCR approaches, that eRNAs interacted with the RAD21 and SMC3 subunits of the cohesin complex. Knockdown of eRNAs by siRNA or locked nucleic acid (LNA) resulted in decreased recruitment of cohesin to those enhancers as determined by RAD21 ChIP, thus providing one of the early demonstrations of a functional role for eRNA. A later study found that RNA is able to stabilize some enhancer-promoter loops. RNAse H1 digestion of RNA prevents RAD21 association with chromatin, thus implicating RNA-RAD21 interactions in formation and stabilization of promoter-enhancer loops (Pezone et al., 2019).

Similarly, the Evf2 eRNA is transcribed from an ultra-conserved enhancer (UCE) region that helps regulate enhancer interactions in *trans* and long-range *cis* (over 31 Mb) in vertebral interneurons. 4C-seq and ChIP-seq (cohesin subunits SMC1 and SMC3) analyses between Evf2+ and Evf2− knockout mouse ganglionic eminences demonstrated that Evf2 regulates cohesin positioning to direct long-range UCE interactions (Cajigas et al., 2018). Additionally, the 5′ UCE-containing region of Evrf2 was sufficient for localizing cohesin to target genes, while the 3′ end was necessary for target gene activation. Although specific to a network of unique UCEs in neurons, Evf2 nevertheless demonstrates the ability of an eRNA to guide cohesin localization and promoter-enhancer formation. Indeed, a similar pattern was observed with the ^DRR^eRNA, which is necessary for muscle cell differentiation. Distinct regions of ^DRR^eRNA were shown to interact with SMC3. ^DRR^eRNA is necessary for the recruitment and localization of cohesin in undifferentiated muscle cells, and its depletion reduces chromatin accessibility and cell differentiation (Tsai et al., 2018).

Several subsequent studies have shown decreases in promoter-enhancer chromatin looping with knockdown of the respective eRNA (Pnueli et al., 2015; Liang et al., 2016; Yang et al., 2016; Tan et al., 2019). In addition to cohesin, heterogeneous nuclear ribonucleoprotein U (hnRNPU) and Mediator complex have also been implicated in eRNA mediated enhancer-promoter looping (Hsieh et al., 2014; Lai et al., 2015; Pnueli et al., 2015; Jiao et al., 2018; Tan et al., 2019). Expression of oncogenic heparanase (HPSE) is controlled by a super enhancer and its cognate eRNA (HPSE eRNA). HPSE eRNA interacts with hnRNPU, increases the binding between hnRNPU and P300, enriches P300 in the super enhancer, and thus promotes chromatin looping between the super enhancer and the HPSE promoter. Overexpression of HPSE eRNA promoted while knockdown decreased this looping interaction and expression of HPSE (Jiao et al., 2018). The Mediator coactivator complex has also been shown to interact specifically with eRNAs and occupies promoter-enhancer looping sites. eRNA knockdown resulted in decrease looping and decreased occupancy of Mediator at target promoters (Lai et al., 2013; Hsieh et al., 2014). An additional complex of interest in eRNA-mediated promoter-enhancer looping is Integrator. Integrator can promote enhancer activation, and loss of its nuclease activity prevents eRNA release from RNAP II. This in turn led to a loss of promoter-enhancer looping and enhancer function (Lai et al., 2015; Barbieri et al., 2018). Finally, Polycomb PRC2 has also been shown to interact with Mediator, maintain looping in poised enhancers, and bind nascent RNAs (Kaneko et al., 2013; Fukasawa et al., 2015; Cruz-Molina et al., 2017). The impact of RNA on methylation of H3K27 by PRC2 is established (as discussed below), but how eRNA may interact with epigenetic modifiers such as PRC2 to promote or maintain looping during transition from poised to active enhancers merits further investigation.

In contrast, other studies have found that enhancer-promoter architecture was maintained in spite of eRNA knockdown or treatment with the RNAP II inhibitor flavopiridol to prevent eRNA transcription (Hah et al., 2013; Schaukowitch et al., 2014). In another study, a polyadenylation termination signal was inserted 80 bp downstream of the Lockd eRNA preventing production of the full eRNA. Notwithstanding, transcription of the target gene CDK91b and promoter-enhancer looping were not altered (Paralkar et al., 2016). There are several potential explanations for these conflicting studies. Some basal level eRNA transcription can occur and ongoing eRNA transcription may not be necessary during mRNA transcription (Rahman et al., 2017). Thus, it may be that enhancer-promoter interactions were established and maintained at these loci before eRNA knockdown. It may also be that eRNA at these loci are not essential for looping, play other roles, or are non-functional. Clearly, eRNAs can interact with necessary DNA looping machinery such as the cohesin and mediator complexes, but the circumstances and mechanisms through which they stabilize, recruit, or guide such complexes to their respective targets remains largely unknown.

#### Modifying Chromatin Accessibility

Most eRNAs are chromatin-associated, as opposed to free in the nucleoplasm or the cytoplasm (Cinghu et al., 2017; Shii et al., 2017). It may therefore be that eRNAs play important roles through their interactions with chromatin-associated proteins. Indeed, knockdown of eRNA has been shown to decrease the accessibility of their respective enhancer regions (as measured by DNase-seq or ATAC-seq), suggesting a role for eRNAs in creating or maintaining open chromatin (Mousavi et al., 2013; Tsai et al., 2018). eRNA has been shown to impact various histone marks (Shii et al., 2017), but its most prominent impact appears to be in modulating acetylation and methylation of H3K27.

The CBP and p300 histone acetyltransferases have been shown to interact with eRNAs (Bose et al., 2017; Jiao et al., 2018), and eRNA knockdown results in decreased H3K27ac at its respective enhancer and target-promoter regions (Pnueli et al., 2015; Liang et al., 2016; Bose et al., 2017; Shii et al., 2017). Bose et al. (2017) demonstrated that eRNAs interact with an RNA binding region within the activation loop of the CBP HAT domain. eRNA displaced the activation loop from the catalytic site and increased the affinity of CBP for its histone substrates. However, a subsequent report challenged these findings as they were unable to reproduce them when looking at p300 unless p300 was purified in buffer containing EDTA (Ortega et al., 2018). Further studies will therefore be needed to clarify if these results represent unique activation mechanisms for p300 and CBP vs. experimental artifact.

Enhancer RNA not only increases H3K27ac but also decreases transcriptionally repressive H3K27me3 levels (Pnueli et al., 2015). This may occur through interactions with the PRC2 complex, which represses transcription through addition of H3K27me3 (Cao et al., 2002). The EZH2 and SUZ12 subunits of PRC2 bind nascent RNA promiscuously with the EZH2 subunit binding to a guanine quadruplex motif in RNA (Kaneko et al., 2013; Wang et al., 2017). PRC2 binding of nascent RNA antagonizes its nucleosome binding activity and protects active gene transcription by inhibiting the methyltransferase activity of PRC2 (Cifuentes-Rojas et al., 2014; Beltran et al., 2016). The eRNA CARMEN has been shown to interact with EZH2 and SUZ12 to alter cardiac precursor cell development and differentiation through epigenetic regualtaion (Ounzain et al., 2015). These studies, in conjunction with the previously discussed studies showing the importance of H3K27ac and the H3K27me2/3 demethylase Kdm6a/b to eRNA production (Li et al., 2017; Kyzar et al., 2019), suggest that eRNAs promote a feed-forward loop of eRNA production and enhancer activation. eRNA production is stimulated by demethylases like Kdm6a/b (Li et al., 2017; Kyzar et al., 2019), and nascent eRNAs protect H3K27 from repressive methylation (Pnueli et al., 2015) and recruit and activate acetyltransferases to promote H3K27 hyperacetylation (Bose et al., 2017; Jiao et al., 2018). This leads to BRD4 recruitment and stabilization (Rahnamoun et al., 2018), additional eRNA production (Kanno et al., 2014), and enhancer activation.

#### Regulation of Transcriptional Machinery

In addition to promoter-enhancer looping and histone modifications, eRNAs interact with transcriptional machinery such as transcription factors, BRD4, and RNAP II. eRNA knockdown decreases binding of transcription factors at respective promoter and enhancer loci, perhaps due to loss of the “transcription factor trapping” mechanism previously discussed (Sigova et al., 2015; Spurlock et al., 2017; Huang Z. et al., 2018). Additionally, the bromodomains of BRD4 have been shown to directly interact with eRNA. This interaction strengthens the affinity of BRD4 for acetylated histones, increases BRD4 and RNAP II recruitment, and promotes transcription (Kanno et al., 2014; Rahnamoun et al., 2018). eRNAs can also recruit RNAP II to both enhancer and promoter loci independently of transcription factor binding to the enhancer (Lai et al., 2013; Mousavi et al., 2013; Maruyama et al., 2014; Yang et al., 2016). Once recruited, eRNA interacts with the NELF and P-TEFb complexes to regulate pause-release of RNAP II and promote enhancer and gene transcription (Schaukowitch et al., 2014; Shii et al., 2017). Kinetically, eRNA first interacts with the p-TEFb subunit CDK9, which phosphorylates NELF and serine 2 of the RNAP II CTD – a critical step for elongation (Shii et al., 2017). Next, eRNA is able to bind to the NELF-E subunit and outcompete nascent mRNA molecules that induce RNAP II stalling when binding NELF-E (Schaukowitch et al., 2014). This leads to eviction of NELF from chromatin and RNA elongation (Schaukowitch et al., 2014; Shii et al., 2017). This mechanism was demonstrated *in vivo* in rat amygdala as LNA knockdown of *Arc* eRNA lead to decreased *Arc* expression and increased NELF-E occupancy at the *Arc* promoter (Kyzar et al., 2019). The binding of eRNA to NELF-E is believed to be non-specific and mediated by promoter-enhancer looping bringing the eRNA into close proximity with the transcriptional machinery. One complex that may contribute to this is Integrator. Integrator has been shown to interact with NELF and DSIF pausing machinery at the proximal promoter and to impact transcription initiation and RNAP II pausing (Gardini et al., 2014; Skaar et al., 2015). In conjunction with its role in eRNA cleavage and maturation, Integrator is uniquely positioned to promote productive eRNA interactions with transcriptional machinery (Lai et al., 2015). Importantly, NELF eviction is the only demonstrated mechanism that directly implicates eRNAs in enhancer mechanism and the production of its respective mRNA.

### Assembly of Super Enhancers

Super enhancers are a recently defined enhancer structure; they occupy a large genomic segment in which dense clusters of enhancers are brought into close 3D proximity and collaborate to act as a single regulatory unit that drives high levels of gene transcription (Hnisz et al., 2013; Whyte et al., 2013; Hah et al., 2015; Sabari et al., 2018). As compared to typical enhancers, super enhancers are marked by dramatically increased levels of transcription factor occupancy; high levels of H3K4me1 and H3K27ac density; increased DNase I hypersensitivity; RNAP II occupancy; and Mediator, p300/CBP, cohesin, and BRD4 cofactor occupancy (Box 2) (Hnisz et al., 2013; Whyte et al., 2013; Witte et al., 2015). Super enhancers are themselves transcribed and produce high quantities of eRNA relative to typical enhancers (Hah et al., 2015; Alvarez-Dominguez et al., 2017). This may be due to very efficient release of RNAP II from NELF-induced pausing at super enhancers in conjunction with decreased reliance on NELF for RNAP II stability (Henriques et al., 2018). One study found that 92% of super enhancers were occupied by RNAP II compared to only 19% of typical enhancers (Pulakanti et al., 2013). Another study reported that super enhancer regions produced on average 26× the number of RNA reads of typical enhancers (Hnisz et al., 2013). Similar to eRNA, super enhancers are associated with lineage-determining genes and play crucial roles in differentiation and cell-specific functions (Hnisz et al., 2013; Whyte et al., 2013; Adam et al., 2015). In many, but not all, super enhancer clusters, individual enhancers appear to exist in a hierarchy from “mother” or “hub” enhancers that organize transcription machinery to a network of additional enhancers in the region (Shin et al., 2016; Huang J. et al., 2018). The spatial layout and intervening DNA may also contribute to super enhancer function. For example, palindromic DNA surrounding the enhancers within the IgH 3′ regulatory region super enhancer provided necessary packaging that contributed to super enhancer structure and functional anatomy (Le Noir et al., 2017). The importance of such intervening DNA sequences has not been explored at other super enhancer loci, thus additional studies will be needed to understand the generalizable role of intervening DNA sequences in super enhancers. Super enhancers are fluid structures that rapidly form and dissolve in response to extracellular stimuli (Gosselin et al., 2014); transcription factors such as NF-κB are able to decommission and establish new super enhancers in response to extracellular stimuli by redistributing necessary super enhancer cofactors such as Mediator and BRD4 (Adam et al., 2015; Schmidt et al., 2015). Thus, both the cofactors (such as BRD4 and Mediator) and the primary DNA structure of super enhancer regions (consisting of individual hierarchical enhancer elements and potentially their unique spacing by intervening DNA sequences) contribute to the final functional 3D structure of super enhancers.

**BOX 2. Defining super enhancers**

Super enhancers are bioinformatically defined by having an exceptionally high concentration of enhancer markers such as cofactors, Mediator, and H3K27ac (Whyte et al., 2013). Traditionally, Med1, H3K27ac, and p300 have been used, although other factors such as BRD4 can be used and a similar profile is obtained (Whyte et al., 2013; Witte et al., 2015; Xiao et al., 2018). ChIP-seq data for H3K27ac (or other marker) is aligned to a reference genome and a peak calling algorithm defines regions of enrichment. Regions within close proximity of each other (12.5 kb as originally described) are stitched together and defined as a single region. All regions are then sorted and ranked according to signal strength (defined as rpm/bp). A graph is produced with the regions sorted by rank along the *X*-axis and signal strength in the *Y*-axis which produces an exponential curve **(A)**. A line is drawn from the highest ranked enhancer to the lowest **(B)**. That line is then slid along the curve until it is tangential to the curve **(C)**. All regions ranked above this point are considered super enhancers, while those below are defined as typical enhancers **(D)**.

Sequencing of nascent RNA can be used to identify and discriminate between typical and super enhancers, similar to how ChIP-seq data has traditionally been used to identify typical and super enhancers (Box 2) (Henriques et al., 2018). Accumulating evidence suggests that eRNAs are not only associated with super enhancers, but may also play a functional role in super enhancer biology (Witte et al., 2015; Aune et al., 2017; Le Gras et al., 2017). eRNAs are known to interact with many of the critical components of super enhancers such as Med1, cohesin, p300/CBP, and BRD4. Many eRNAs derived from super enhancers have been shown to play biological roles both with respective to their host super enhancer and at distant loci, and may promote interactions between super enhancers and target regions (Ounzain et al., 2015; Pefanis et al., 2015; Witte et al., 2015; Liang et al., 2016; Alvarez-Dominguez et al., 2017; Spurlock et al., 2017; Jiao et al., 2018; Tsai et al., 2018). shRNA knockdown of the eRNA from an Epstein-Barr Virus-induced super enhancer led to decreased H3K27ac, decreased looping between the super enhancer and the target MYC locus, and decreased MYC expression (Liang et al., 2016). Dysregulation of eRNA from super enhancers correlated with downregulation of respective striatal neuron identity genes in a mouse model of Huntington’s disease. This eRNA dysregulation was associated with decreased H3K27ac and a loss of RNAP II binding sites, suggesting the importance of these marks to both eRNA and super enhancer function (Le Gras et al., 2017). Similarly, a genome-wide association study found that differential eRNA expression in patients with autoimmune disease was especially localized to super enhancers near known genetic variants for autoimmune disease risk (Aune et al., 2017). Thus, eRNA appears to play an important role in super enhancer biology.

### Enhancers, eRNA, and Phase Separation

**FIGURE 3:**
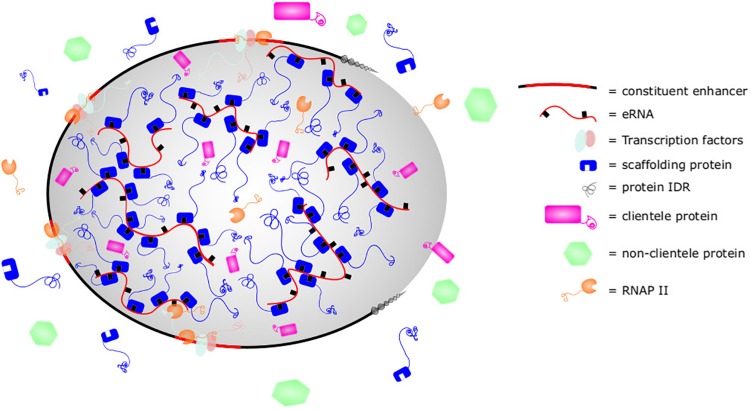
Super enhancer as phase-separated structure. Phase separated structures such as super enhancers are composed of scaffolding nucleic acids and proteins with intrinsically disordered regions (IDRs). Thermodynamically, they are favored to condense together at greater concentration than found in the cytoplasm, leading to formation of a membraneless phase separated structure. This allows the concentration of clientele proteins with similar biophysical properties and exclusion of other dissimilar proteins. By altering the stoichiometry of scaffolding or clients, cells can control the makeup of biomolecular condensates. In super enhancers, eRNA sequence motifs, secondary structures, or post-translational modifications such as m6A may be bound by Med1, BRD4, or other proteins with IDRs to promote organization, structure, and function of super enhancers.

Recent studies have shown that super enhancers exist as liquid-liquid phase separated biomolecular condensates (Sabari et al., 2018) (Figure 3; see Box 1 for description of phase separation). Similarly, enhancers activated by the MegaTrans complex of transcription factors (MegaTrans enhancers) were shown to undergo phase separation (Liu et al., 2014; Nair et al., 2019). The activation domain of some transcription factors are also known to mediate phase separation (Boija et al., 2018). Recent evidence supporting the role of eRNA in phase separation and enhancer function was demonstrated with the MegaTrans complex. Using a combination of ChIP, fluorescently labeled IVT eRNAs, ASO, and FISH, Nair et al. (2019) showed that eRNAs from MegaTrans enhancers form enhancer ribonucleoprotein (eRNP) complexes with condensins. These eRNPs are necessary for phase separation and MegaTrans enhancer function. This is the first demonstration of eRNA playing a direct role in phase separation and enhancer function. eRNA may contribute to enhancer phase separation as a scaffolding member that is necessary for organizing phase separation and/or as a client member which participates in the structure and function (Banani et al., 2016). We will review evidences demonstrating how eRNA could impact phase separation as a scaffolding or client member. As phase separation may not be limited to defined “super enhancers” and may include other strongly activating enhancers, we will refer to all such enhancers as “phase separated enhancers” (PSE) in recognition that the following studies are applicable to but may not be limited to super enhancers.

RNA in general is known to act as a scaffold and directly contribute to the formation of many phase-separated structures. For example, competition for mRNA drives the formation and dissolution of P granules, while the lncRNA NEAT1 provides a scaffold for the formation of paraspeckles (Saha et al., 2016; Fox et al., 2018). Similarly, mRNA is able to phase separate cytosolic proteins when multiple m^6^A methylations are present. The m^6^A marks serve as binding platforms for YTHDF proteins and arranges their IDRs to induce phase separation (Ries et al., 2019). Interestingly, this same m^6^A modification is enriched in eRNA (Xiao et al., 2019). eRNA is known to interact with proteins such as Med1 and BRD4, the IDRs of which contribute to super enhancer formation through phase separation (Sabari et al., 2018). Proteins in the MegaTrans complex are also enriched in IDRs (Nair et al., 2019). Thus, PSEs are enriched with proteins with IDRs and eRNA may serve as a scaffold for the induction of phase separation by interacting with RBDs in these proteins’ IDRs.

In addition to contributing to PSE formation, eRNA may also serve as a potential client and contribute to the composition, biophysical characteristics, and maturation of PSEs. For example, HPSE eRNAs interact with and recruit both hnRNPU and P300 to super enhancers (Jiao et al., 2018). Other hnRNPs, such as hnRNPA1, have been shown to undergo phase separation through IDRs, RBDs, and RNA (Lin et al., 2015; Molliex et al., 2015; Maharana et al., 2018; Wang et al., 2018), and the recruitment of hnRNPU or P300 to super enhancers by eRNA could model the ability of phase separated structures to fuse when in proximity. Intriguingly, the CTD of RNAP II is able to be recruited into biomolecular condensates (Cho et al., 2018). Its specific phosphorylation states affect its preferential incorporation into transcription initiation or splicing condensates (Guo et al., 2019). Such a model may also explain how RNAP II is recruited to super enhancers. This would explain the findings in studies showing eRNA playing a role in RNAP II recruitment to the MyoD super enhancer and other loci if eRNA were important to the phase separation (Mousavi et al., 2013; Maruyama et al., 2014). eRNA may also contribute to PSE maturation, as RNA is known to play important roles in maturation of many molecular condensates. The importance of condensate maturation to PSEs was demonstrated in MegaTrans enhancers where acutely activated enhancers were susceptible to chemical disruption of phase separation whereas chronically activated enhancers were resistant and assumed a more “gel-like” state (Nair et al., 2019). eRNA may therefore influence the composition of proteins recruited, the biophysical properties, and the stability or maturation of PSEs.

The high production of eRNA by PSEs, the pervasive interactions between eRNA and PSE components, and the critical role of RNAs in biomolecular condensate biology all suggest a critical relationship between eRNA and PSEs. These ideas were captured in the discovery of eRNPs as essential contributors to MegaTrans enhancers, and analogous complexes await discovery in super enhancers or other PSEs. Accordingly, outstanding questions regarding the formation, regulation, maintenance, dissolution, and longevity of super enhancers may find answers in eRNAs and their impacts on super enhancers as biomolecular condensates. Thus, the impact of eRNA on PSEs is a potentially significant biological role for eRNA. Future research will shed light on the role of eRNA as scaffold or client in PSEs and the impact such roles have on enhancer biology.

## eRNAs as Markers of Active Enhancers

Because of their biological roles in gene transcription, eRNAs serve as markers of active enhancers. eRNA transcription *per se* is able to identify *in vivo* enhancers that are “caught in the act” of regulating mRNA/gene expression and does so independently of the local chromatin state or transcription factor binding (Kim et al., 2010; Wang et al., 2011; Sanyal et al., 2012; Hah et al., 2013; Andersson et al., 2014a; Wu et al., 2014; Arner et al., 2015; Cheng et al., 2015; Ren et al., 2017; Imamura et al., 2018). For example, one study found that eRNA served as a better marker of active enhancers than H3K27ac (Tyssowski et al., 2018). The ability to mark active enhancers in cells is an opportunity for unique insights, and several studies have begun to successfully utilize eRNA to mark active enhancer regions (Wang et al., 2011; Cauchy et al., 2016; Baillie et al., 2017; Denisenko et al., 2017; Henriques et al., 2018). eRNA expression profiles are highly tissue-specific (Koch et al., 2011; Djebali et al., 2012; Arner et al., 2015; Cheng et al., 2015; Ren et al., 2017) and can differentiate between different activation states of cells (Benner et al., 2015; Denisenko et al., 2017). As there are many more enhancer regions than gene loci, utilizing eRNA may provide unique opportunities and advantages for diagnosing, prognosticating, treating, and understanding the pathogenesis of diseases that aren’t afforded by current biomarkers. Multiple genome-wide association studies have found that most disease associated mutations occur outside the coding genome, and that a majority of those occur in enhancers or their transcribed RNAs (Farh et al., 2014; Aune et al., 2017). Accordingly, eRNAs are beginning to be utilized to map specific disease states and show the ability to identify disease-specific variants in a broad range of diseases including autoimmunity, cancer, infectious disease, cardiac hypertrophy, recurrent pregnancy loss, psychiatric, and neurological disorders (Farh et al., 2014; Witte et al., 2015; Liang et al., 2016; Aune et al., 2017; Le Gras et al., 2017; Ren et al., 2017; Hauberg et al., 2018; Huang Z. et al., 2018; Imamura et al., 2018; Jiao et al., 2018; Gu et al., 2019; Kyzar et al., 2019; Mirtschink et al., 2019; Tan et al., 2019). The therapeutic potential of these studies was demonstrated when knockdown of the HERNA1 eRNA, which promotes pathological cardiac hypertrophy, protected mice from disease and reversed pathology in mice that had already developed the disease (Mirtschink et al., 2019). eRNA also provides a means for studying the dysregulation of mRNA in cancers and other diseases, as specific eRNAs have also been shown to hold prognostic value to patients with squamous cell carcinoma of the head and neck (Gu et al., 2019). We anticipate that the study of eRNA will continue to contribute to our understanding of the molecular pathogenesis and treatment of human disease.

## Conclusion and Future Directions

Enhancer RNAs are products of active enhancers and correlate with mRNA transcription of target genes but with unique expression profiles. Although broadly transcribed, eRNAs are a diverse array of RNAs with heterogeneous structure, length, and post-transcriptional modifications. Recent studies showing decreased gene transcription when eRNA is knocked down suggests that eRNAs have important roles in gene regulation and cellular biology. Mechanisms of action of eRNA include enhancer-promoter looping, chromatin modification, and regulation of transcriptional machinery. Importantly, the emergence of PSE such as super enhancers opens new possibilities for a role of eRNA as mediators of phase separation. The recent discovery that phase separation may be the means through which the activation domains of many transcription factors function, in addition to phase separation of MegaTrans enhancers, raises questions about the ubiquity of phase separation at enhancers and whether typical enhancers also utilize phase separation as a means to regulate gene expression. If so, eRNA-mediated phase separation may be the unified model by which various structures and forms of eRNA mediate looping, recruitment, and other biological functions. Future studies looking at eRNA should address whether the eRNA is transcribed from a phase-separated enhancer and if so, whether such eRNA is required for phase separation. Such studies promise to further elucidate the role of eRNA in the control of cell functions and fate decisions.

## Author Contributions

PA researched, developed, wrote the review, and created figures. AW and XL edited, developed, and provided direction to the review.

## Conflict of Interest

The authors declare that the research was conducted in the absence of any commercial or financial relationships that could be construed as a potential conflict of interest.
