# Supplementary material for: Diversity and Emerging Roles of Enhancer RNA in Regulation of Gene Expression and Cell Fate
Source: Front Cell Dev Biol. 2020 Jan 14;7:377. doi: 10.3389/fcell.2019.00377 (PMC6971116; doi:10.3389/fcell.2019.00377)
Supplement: Supplementary file 1 [file Table_1.DOCX]

| Supplementary Table 1: eRNAs shown to exhibit biological activity, mechanism of action if known, and methods used to determine biological function. | | | | | |
| --- | --- | --- | --- | --- | --- |
| eRNA | Target Gene | Method | Notes on eRNA | Mechanism of action | Ref |
| IL1β-eRNA | IL1β  CXCL8 | LNA* knockdown | Upregulated in response to LPS in human primary monocytes. Regulated by NF-κB. | Unknown | (NE et al., 2014) |
| IL1β-RBT46 | IL1β  CXCL8 | LNA (both + and - strands) knockdown | Upregulated in response to LPS in human primary monocytes. Regulated by NF-κB. | Unknown | (NE et al., 2014) |
| Gadd45b eRNA | Growth arrest and DNA-damage-inducible, beta | shRNA (+ strand) knockdown | Induced by membrane depolarization in primary mouse cortical neurons. Does not affect looping. | Facilitates NELF release during gene activation | (Schaukowitch et al., 2014) |
| Arc eRNA | Activity-regulated cytoskeletal protein (Arc) | shRNA and LNA (- strand) knockdown in vitro and in vivo | Induced by membrane depolarization in primary mouse cortical neurons. Does not affect looping. | Facilitates NELF release during gene activation | (Schaukowitch et al., 2014; Kyzar et al., 2019) |
| 774 and 775 | SERPINB2 (plasminogen activator inhibitor-2, (PAI-2)) | ASO** knockdown  Vector overexpression | Upregulated in response to LPS in MonoMac6 human monocyte cell line. | Alters chromatin modifications and interacts with CDK9 to promote NELF eviction and release of RNAP II pausing | (Shii et al., 2017) |
| HPSE eRNA | Heparanase (HPSE) | shRNA knockdown  Ectopic overexpression | Super enhancer derived. Promotes cancer progression in multiple cell lines. Can serve as independent prognostic of poor outcome in cancer patients. | Binds hnRNPU to promote chromatin looping | (Jiao et al., 2018) |
| Cx3cr1 eRNA | Cx3cr1 | siRNA and ASO knockdown (anti-sense strand), in vitro enhancer assay | Mouse macrophages. Rev-Erb may regulate regions by repressing eRNA expression. | Unknown | (Lam et al., 2013) |
| Mmp9 eRNA | Mmp9 | siRNA and ASO knockdown (sense strand), in vitro enhancer assay  shRNA | Mouse macrophages and human SW480 and 293T cells. Upregulated in response to TNF-α. Rev-Erb may regulate regions by repressing eRNA expression. In vivo knockdown reduced Mmp9 demonstrating potential therapeutic potential in mouse model. | May interact with BRD4 to increase BRD4 binding to acetylated histones | (Lam et al., 2013; Rahnamoun et al., 2018) |
| CCL2 eRNA | CCL2 | shRNA | Human SW480 and 293 T cells. Upregulated in response to TNF-α. | May interact with BRD4 to increase BRD4 binding to acetylated histones | (Rahnamoun et al., 2018) |
| C/EBPβ eRNA | CCAAT/enhancer-binding protein-beta (C/EBPβ) | siRNA | Expression tied to enhancer hypomethylation. Expressed in human hepatocellular carcinoma and linked with tumor progression. Expression reinforced by its gene product (C/EBPβ). | Unknown | (Xiong et al., 2019) |
| ^DRR^eRNA | Myogenin | siRNA | Expressed from MyoD super enhancer, but regulates Myogenin in *trans* in C2C12 cells (myoblast) and myotubes. | Specific domains interact with SMC3 to recruit and maintain Cohesin at the myogenin locus. Establishes promoter/enhancer looping. | (Mousavi et al., 2013; Tsai et al., 2018) |
| ^CE^RNA | MYOD1 | siRNA | Derived from same super enhancer locus as ^DRR^eRNA. | Increases chromatin accessibility | (Mousavi et al., 2013) |
| Cga eRNA | Chorionic gonadotropin alpha | shRNA targeting negative strand | Prolonged culture of murine gonadotrope cell line with shRNA led to increasing repression of Cga. Altered histone modifications. | Promoter/enhancer looping, Histone modifications | (Pnueli et al., 2015) |
| KLK3e | Kallikrein-related peptidase 3 (KLK3/PSA) and KLK2. | siRNA of sense strand | Bidirectionally transcribed, but forward strand carries greater biological activity. | Promoter/enhancer looping | (Hsieh et al., 2014) |
| Bloodlinc | Band3 in cis, multiple others (488) in trans. | shRNA and ectopic overexpression | Acts in trans to promote erythropoiesis. Interacts with HNRNPU protein. Super enhancer derived. | Unknown. May act as scaffold. | (Alvarez-Dominguez et al., 2014; Alvarez-Dominguez et al., 2017) |
| Pfkl-eRNA | Pfkl | LNA | Undergoes cytosine methylation by NSun7. | Unknown | (Aguilo et al., 2016) |
| Sirt5-eRNA | Sirt5 | LNA and doxycycline-inducible trans-activation model | Undergoes cytosine methylation by NSun7. | Unknown | (Aguilo et al., 2016) |
| p53BER2-eRNA | PAPPA | siRNA and luciferase reporter assays | Necessary to p53-mediated gene expression. Decreased RNAPII binding after knockdown. Specific region of eRNA critical for function. | Unknown | (Melo et al., 2013) |
| p53BER4 | IER5 | siRNA | Necessary to p53-mediated gene expression. Decreased RNAPII binding after knockdown. | Unknown | (Melo et al., 2013) |
| Zmynd8as | Zmynd8 | ASO | Knockdown altered isoform expression. | Unknown | (Onodera et al., 2012) |
| FOXC1e | FOXC1 | siRNA/LNA and GAL4-BoxB-tethering-based reporter assay | Induced by E2 (17β-oestradiol) binding in MCF-7 human breast cancer cells. Interacts with SMC3 and RAD21 to recruit Cohesin to promoter. | Suspected promoter/enhancer looping | (Li et al., 2013) |
| TFF1e | TFF1 | siRNA/LNA | Induced by E2 (17β-oestradiol) binding in MCF-7 human breast cancer cells. Interacts with SMC3 and RAD21 to recruit Cohesin to promoter. | Suspected promoter/enhancer looping | (Li et al., 2013) |
| CA12e | CA12 | siRNa/LNA | Induced by E2 (17β-oestradiol) binding in MCF-7 human breast cancer cells. | Unknown | (Li et al., 2013) |
| NRIP1e | NRIP1 | LNA | Induced by E2 (17β-oestradiol) binding in MCF-7 human breast cancer cells. Interacts with SMC3 and RAD21 to recruit Cohesin to promoter. | Promoter/enhancer looping | (Li et al., 2013) |
| GREB1e | GREB | siRNA | Induced by E2 (17β-oestradiol) binding in MCF-7 human breast cancer cells. | Promoter/enhancer looping | (Li et al., 2013) |
| PGRe | PGR | siRNA | Induced by E2 (17β-oestradiol) binding in MCF-7 human breast cancer cells. | Unknown | (Li et al., 2013) |
| KCNK5e | KCNK5 | siRNA | Induced by E2 (17β-oestradiol) binding in MCF-7 human breast cancer cells. | Unknown | (Li et al., 2013) |
| P2RY2e | P2RY2 | siRNA | Induced by E2 (17β-oestradiol) binding in MCF-7 human breast cancer cells. Interacts with SMC3 and RAD21. | Suspected promoter/enhancer looping | (Li et al., 2013) |
| SMAD7e | SMAD7 | siRNA | Induced by E2 (17β-oestradiol) binding in MCF-7 human breast cancer cells. | Unknown | (Li et al., 2013) |
| SIAH2e | SIAH2 | siRNA | Induced by E2 (17β-oestradiol) binding in MCF-7 human breast cancer cells. | Unknown | (Li et al., 2013) |
| Plekhf2e | Plekhf2 | ASO | Down-regulated in response to ERα binding in MCF-7 human breast cancer cells. | Enhancer/promoter looping | (Tan et al., 2018) |
| Sytl2e | Sytl2 | ASO | Down-regulated in response to ERα binding in MCF-7 human breast cancer cells. | Enhancer/promoter looping | (Tan et al., 2018) |
| ncRNA-a3 | TAL1 | siRNA  luciferase reporter | Recruits Med and RNAP II to enhancer region, promotes Med H3 kinase activity. | Promoter/enhancer looping | (Orom et al., 2010; Lai et al., 2013) |
| ncRNA-a7 | SNAI1 and AURKA | siRNA  luciferase reporter | Recruits Med and RNAP II to enhancer region, promotes Med H3 kinase activity. | Promoter/enhancer looping | (Ørom et al., 2010; Lai et al., 2013) |
| ncRNA-a1 | ECM1 | siRNA |  | Unknown | (Ørom et al., 2010) |
| ncRNA-a2 | KLHL12 | siRNA |  | Unknown | (Ørom et al., 2010) |
| ncRNA-a4 | CMPK1 | siRNA  luciferase reporter |  | Unknown | (Ørom et al., 2010) |
| ncRNA-a5 | ROCK2 | siRNA  luciferase reporter |  | Unknown | (Ørom et al., 2010) |
| ncRNA-a6 | Snai2 | siRNA |  | Unknown | (Ørom et al., 2010) |
| MYC-428 ESE eRNA | MYC | shRNA | From super enhancer induced by EBV. Promotes cancer cell line growth and H3K27ac at enhancer. | Histone modification and promoter/enhancer looping | (Liang et al., 2016) |
| MYE-525 ESE eRNA | MYC | shRNA | From super enhancer induced by EBV. Promotes cancer cell line growth and H3K27ac at enhancer. | Histone modification and promoter/enhancer looping | (Liang et al., 2016) |
| eRNA-YY1 | YY1 | ASO | Interacts with regulatory domain of CBP. | Promotes H3K27ac by CBP | (Raisner et al., 2018) |
| eRNA-Ccnd1 | Ccnd1 | ASO | Interacts with regulatory domain of CBP. | Promotes H3K27ac by CBP | (Raisner et al., 2018) |
| S-IRE1 eRNA | Ifnb1 | siRNA | Expressed from sumoylated enhancer upstream of Ifnb1. Decreased sumoylation increases expression in response to LPS. | Unknown | (Decque et al., 2016) |
| L2 eRNA | Ifnb1 | siRNA (+ and – strands) |  | Uknown | (Banerjee et al., 2014) |
| eRNA E2-3 | HO-1 | siRNA | Induced by oxidative stress. Positively regulated by NRF2, and negatively regulated by BACH1. | Alters RNAP II binding to promoter and enhancer regions | (Maruyama et al., 2014) |
| AS1eRNA | DHRS4-AS1 | siRNA | Promotes RNAP II and P300 binding to promoter and enhancer regions. | Enhancer/promoter looping | (Yang et al., 2016) |
| Lnc-LSC4A1-1 | CXCL8 | siRNA and RNA-IP | Recruits NF-kB to the CXCL8 locus. Promotes unexplained recurrent pregnancy loss. | Transcription factor trapping | (Huang et al., 2018) |

*LNA: locked nucleic acid anti-sense inihibitors

**ASO: antisense oligonucleotide

**References**

Aguilo, F., Li, S., Balasubramaniyan, N., Sancho, A., Benko, S., Zhang, F., et al. (2016). Deposition of 5-Methylcytosine on Enhancer RNAs Enables the Coactivator Function of PGC-1α. *Cell Reports* 14(3)**,** 479-492. doi: <https://doi.org/10.1016/j.celrep.2015.12.043>.

Alvarez-Dominguez, J.R., Hu, W., Yuan, B., Shi, J., Park, S.S., Gromatzky, A.A., et al. (2014). Global discovery of erythroid long noncoding RNAs reveals novel regulators of red cell maturation. *Blood* 123(4)**,** 570-581. doi: 10.1182/blood-2013-10-530683.

Alvarez-Dominguez, J.R., Knoll, M., Gromatzky, A.A., and Lodish, H.F. (2017). The Super-Enhancer-Derived alncRNA-EC7/Bloodlinc Potentiates Red Blood Cell Development in trans. *Cell Rep* 19(12)**,** 2503-2514. doi: 10.1016/j.celrep.2017.05.082.

Banerjee, A.R., Kim, Y.J., and Kim, T.H. (2014). A novel virus-inducible enhancer of the interferon-β gene with tightly linked promoter and enhancer activities. *Nucleic Acids Research* 42(20)**,** 12537-12554. doi: 10.1093/nar/gku1018.

Decque, A., Joffre, O., Magalhaes, J.G., Cossec, J.C., Blecher-Gonen, R., Lapaquette, P., et al. (2016). Sumoylation coordinates the repression of inflammatory and anti-viral gene-expression programs during innate sensing. *Nat Immunol* 17(2)**,** 140-149. doi: 10.1038/ni.3342.

Hsieh, C.L., Fei, T., Chen, Y., Li, T., Gao, Y., Wang, X., et al. (2014). Enhancer RNAs participate in androgen receptor-driven looping that selectively enhances gene activation. *Proc Natl Acad Sci U S A* 111(20)**,** 7319-7324. doi: 10.1073/pnas.1324151111.

Huang, Z., Du, G., Huang, X., Han, L., Han, X., Xu, B., et al. (2018). The enhancer RNA lnc-SLC4A1-1 epigenetically regulates unexplained recurrent pregnancy loss (URPL) by activating CXCL8 and NF-kB pathway. *EBioMedicine*. doi: <https://doi.org/10.1016/j.ebiom.2018.11.015>.

Jiao, W., Chen, Y., Song, H., Li, D., Mei, H., Yang, F., et al. (2018). HPSE enhancer RNA promotes cancer progression through driving chromatin looping and regulating hnRNPU/p300/EGR1/HPSE axis. *Oncogene* 37(20)**,** 2728-2745. doi: 10.1038/s41388-018-0128-0.

Kyzar, E.J., Zhang, H., and Pandey, S.C. (2019). Adolescent Alcohol Exposure Epigenetically Suppresses Amygdala Arc Enhancer RNA Expression to Confer Adult Anxiety Susceptibility. *Biol Psychiatry* 85(11)**,** 904-914. doi: 10.1016/j.biopsych.2018.12.021.

Lai, F., Orom, U.A., Cesaroni, M., Beringer, M., Taatjes, D.J., Blobel, G.A., et al. (2013). Activating RNAs associate with Mediator to enhance chromatin architecture and transcription. *Nature* 494(7438)**,** 497-501. doi: 10.1038/nature11884.

Lam, M.T., Cho, H., Lesch, H.P., Gosselin, D., Heinz, S., Tanaka-Oishi, Y., et al. (2013). Rev-Erbs repress macrophage gene expression by inhibiting enhancer-directed transcription. *Nature* 498(7455)**,** 511-515. doi: 10.1038/nature12209.

Li, W., Notani, D., Ma, Q., Tanasa, B., Nunez, E., Chen, A.Y., et al. (2013). Functional roles of enhancer RNAs for oestrogen-dependent transcriptional activation. *Nature* 498(7455)**,** 516-520. doi: 10.1038/nature12210.

Liang, J., Zhou, H., Gerdt, C., Tan, M., Colson, T., Kaye, K.M., et al. (2016). Epstein-Barr virus super-enhancer eRNAs are essential for MYC oncogene expression and lymphoblast proliferation. *Proc Natl Acad Sci U S A* 113(49)**,** 14121-14126. doi: 10.1073/pnas.1616697113.

Maruyama, A., Mimura, J., and Itoh, K. (2014). Non-coding RNA derived from the region adjacent to the human HO-1 E2 enhancer selectively regulates HO-1 gene induction by modulating Pol II binding. *Nucleic Acids Research* 42(22)**,** 13599-13614. doi: 10.1093/nar/gku1169.

Melo, C.A., Drost, J., Wijchers, P.J., van de Werken, H., de Wit, E., Oude Vrielink, J.A., et al. (2013). eRNAs are required for p53-dependent enhancer activity and gene transcription. *Mol Cell* 49(3)**,** 524-535. doi: 10.1016/j.molcel.2012.11.021.

Mousavi, K., Zare, H., Dell'orso, S., Grontved, L., Gutierrez-Cruz, G., Derfoul, A., et al. (2013). eRNAs promote transcription by establishing chromatin accessibility at defined genomic loci. *Mol Cell* 51(5)**,** 606-617. doi: 10.1016/j.molcel.2013.07.022.

NE, I.I., Heward, J.A., Roux, B., Tsitsiou, E., Fenwick, P.S., Lenzi, L., et al. (2014). Long non-coding RNAs and enhancer RNAs regulate the lipopolysaccharide-induced inflammatory response in human monocytes. *Nat Commun* 5**,** 3979. doi: 10.1038/ncomms4979.

Onodera, C.S., Underwood, J.G., Katzman, S., Jacobs, F., Greenberg, D., Salama, S.R., et al. (2012). Gene Isoform Specificity through Enhancer-Associated Antisense Transcription. *PLOS ONE* 7(8)**,** e43511. doi: 10.1371/journal.pone.0043511.

Orom, U.A., Derrien, T., Beringer, M., Gumireddy, K., Gardini, A., Bussotti, G., et al. (2010). Long noncoding RNAs with enhancer-like function in human cells. *Cell* 143(1)**,** 46-58. doi: 10.1016/j.cell.2010.09.001.

Ørom, U.A., Derrien, T., Beringer, M., Gumireddy, K., Gardini, A., Bussotti, G., et al. (2010). Long Noncoding RNAs with Enhancer-like Function in Human Cells. *Cell* 143(1)**,** 46-58. doi: <https://doi.org/10.1016/j.cell.2010.09.001>.

Pnueli, L., Rudnizky, S., Yosefzon, Y., and Melamed, P. (2015). RNA transcribed from a distal enhancer is required for activating the chromatin at the promoter of the gonadotropin alpha-subunit gene. *Proc Natl Acad Sci U S A* 112(14)**,** 4369-4374. doi: 10.1073/pnas.1414841112.

Rahnamoun, H., Lee, J., Sun, Z., Lu, H., Ramsey, K.M., Komives, E.A., et al. (2018). RNAs interact with BRD4 to promote enhanced chromatin engagement and transcription activation. *Nat Struct Mol Biol* 25(8)**,** 687-697. doi: 10.1038/s41594-018-0102-0.

Raisner, R., Kharbanda, S., Jin, L., Jeng, E., Chan, E., Merchant, M., et al. (2018). Enhancer Activity Requires CBP/P300 Bromodomain-Dependent Histone H3K27 Acetylation. *Cell Rep* 24(7)**,** 1722-1729. doi: 10.1016/j.celrep.2018.07.041.

Schaukowitch, K., Joo, J.Y., Liu, X., Watts, J.K., Martinez, C., and Kim, T.K. (2014). Enhancer RNA facilitates NELF release from immediate early genes. *Mol Cell* 56(1)**,** 29-42. doi: 10.1016/j.molcel.2014.08.023.

Shii, L., Song, L., Maurer, K., Zhang, Z., and Sullivan, K.E. (2017). SERPINB2 is regulated by dynamic interactions with pause-release proteins and enhancer RNAs. *Mol Immunol* 88**,** 20-31. doi: 10.1016/j.molimm.2017.05.005.

Tan, Y., Jin, C., Ma, W., Hu, Y., Tanasa, B., Oh, S., et al. (2018). Dismissal of RNA Polymerase II Underlies a Large Ligand-Induced Enhancer Decommissioning Program. *Molecular Cell* 71(4)**,** 526-539.e528. doi: <https://doi.org/10.1016/j.molcel.2018.07.039>.

Tsai, P.F., Dell'Orso, S., Rodriguez, J., Vivanco, K.O., Ko, K.D., Jiang, K., et al. (2018). A Muscle-Specific Enhancer RNA Mediates Cohesin Recruitment and Regulates Transcription In trans. *Mol Cell* 71(1)**,** 129-141.e128. doi: 10.1016/j.molcel.2018.06.008.

Xiong, L., Wu, F., Wu, Q., Xu, L., Cheung, O.K., Kang, W., et al. (2019). Aberrant enhancer hypomethylation contributes to hepatic carcinogenesis through global transcriptional reprogramming. *Nat Commun* 10(1)**,** 335. doi: 10.1038/s41467-018-08245-z.

Yang, Y., Su, Z., Song, X., Liang, B., Zeng, F., Chang, X., et al. (2016). Enhancer RNA-driven looping enhances the transcription of the long noncoding RNA DHRS4-AS1, a controller of the DHRS4 gene cluster. *Scientific Reports* 6**,** 20961. doi: 10.1038/srep20961

<https://www.nature.com/articles/srep20961#supplementary-information>.
